# Reevaluation of *Pholiota squarrosa* lectin-reactive haptoglobin as a pancreatic cancer biomarker using an improved ELISA system

**DOI:** 10.1007/s10719-017-9772-9

**Published:** 2017-04-28

**Authors:** Ken Kusama, Yuki Okamoto, Keiko Saito, Tsukasa Kasahara, Teizo Murata, Yasushi Ueno, Yuka Kobayashi, Yoshihiro Kamada, Eiji Miyoshi

**Affiliations:** 1 J-Oil Mills Inc., 11, Kagetoricho, Totsuka-ku, Yokohama, Kanagawa 245-0064 Japan; 2 Department of Molecular Biochemistry & Clinical Investigation, Osaka University Graduate School of Medicine, Osaka, Japan

**Keywords:** Pancreatic cancer, Fucosylated haptoglobin, CA19–9, Denaturation, Elisa, *Pholiota squarrosa* lectin

## Abstract

An increase in Lewis- and core-type fucosylation of haptoglobin has been reported in patients with pancreatic cancer (PC), suggesting that fucosylated haptoglobin is a candidate PC biomarker. Previously, we developed a *Pholiota squarrosa* lectin antibody enzyme-linked immunosorbent assay (PhoSL-ELISA) system for the detection of core-fucosylated haptoglobin. However, with this methodology, positive results were only obtained for some patients with PC, demonstrating the need for a more sensitive detection system. In the current study, we developed an improved PhoSL-ELISA system with higher sensitivity to detect core-fucosylated haptoglobin using high-concentration urea as a denaturing agent with lectin to facilitate detection. We then reevaluated the performance of PhoSL reactive-core-fucosylated haptoglobin (PhoSL-HP) as a PC biomarker using the improved PhoSL-ELISA system. PhoSL-HP levels in the sera of patients with PC were significantly higher than those in healthy volunteers, with an area under the curve (AUC) value of 0.753. Furthermore, the AUC value of CA19–9 improved from 0.793 to 0.907 when combined with PhoSL-HP. Additionally, several CA19–9-negative cases among the patients with PC were diagnosed as positive for PhoSL-HP. In conclusion, PhoSL-HP detection using our improved ELISA system might allow PhoSL-HP to serve as a potential biomarker for PC and thus might be useful to complement the detection of CA19–9 in PC diagnosis.

**Electronic supplementary material:**

The online version of this article (doi:10.1007/s10719-017-9772-9) contains supplementary material, which is available to authorized users.

## Introduction

Pancreatic cancer is one of the leading causes of cancer deaths. The overall survival is less than 5% [1]. A possible reason for this low survival rate is that detection of pancreatic cancer (PC) is difficult at the early stages of the disease. Carbohydrate antigen 19–9 (CA19–9), carcinoembryonic antigen, and DUPAN-2 are commonly used for detecting PC. However, false positives are often obtained with these markers, which hinder their use for early diagnosis [2]. In addition, although CA19–9 is a commonly used tumor marker for the diagnosis of PC, it cannot be used for the Lewis-negative phenotype; conversely, DUPAN-2 has been reported to be a good marker for the detection of the Lewis-negative phenotype of PC [3]. Therefore, combination analyses using another marker together with CA19–9 are generally conducted to provide a more accurate assessment of PC. Recently, Apo AII protein isoforms were also demonstrated as potential markers for screening for PC [4]. However, Apo AII was not able to distinguish PC from other pancreatic diseases including chronic pancreatitis (CP) and intraductal papillary mucinous neoplasm. Therefore, it is important to identify biomarkers with different characteristics to facilitate the accurate diagnosis of PC either in a single assay or in combination with CA19–9.

Fucosylation, the addition of a fucose group to a protein through an *N*-linked or *O*-linked glycan, can occur through multiple types of glycosidic linkages such as α1–2, α1–3, α1–4, and α1–6. Some types of fucosylation have been shown to be involved in cancer and inflammation [5]. Furthermore, increased fucosylation of Golgi protein 73 was reported to be involved in hepatocellular carcinoma progression [6]. Thus, fucosylated glycoproteins are considered to represent candidate cancer biomarkers.

Haptoglobin is an acute-phase protein produced in the liver that consists of α- and β-subunits linked by inter-chain disulfide bonds. Previous studies found that increased amounts of α1–3, α1–4, and α1–6 fucosylated haptoglobin were present in the sera of patients with PC [7–12]. In addition, glycoprotein microarray analysis showed that significantly more fucosylation occurred in the sera of patients with PC than in those with CP [13]. Haptoglobin has four *N*-glycan sites, each with unique oligosaccharides that can include fucosylated *N*-glycan [8]; in particular, the levels of the di-fucosylated *N*-glycan of haptoglobin were reported to increase following PC-specific modification [14]. Therefore, fucosylated haptoglobin may potentially represent a specific biomarker for PC.

Both *Aleuria aurantia* lectin (AAL) [15] and *Pholiota squarrosa* lectin (PhoSL) [16] have been used to detect fucosylated haptoglobin. We previously found that haptoglobin derived from the PC cell line PSN1 was highly reactive with PhoSL and that the haptoglobin levels detected by PhoSL were increased in the sera of patients with PC compared with healthy volunteers (HVs) [17]. In addition, Matsumoto *et al.* [9] and Kamada *et al.* [10] developed a lectin-enzyme linked immunosorbent assay (ELISA) kit using Lewis- and core-type fucose-binding AAL, and showed that AAL reactive-fucosylated haptoglobin (AAL-HP) might represent a potential biomarker for PC. Similarly, Shimomura *et al.* [11] developed an ELISA kit using core-type fucose-binding PhoSL and demonstrated that PhoSL-HP levels were slightly elevated in patients with PC. More recently, Ueda *et al.* [12] showed that the PhoSL-HP detected with this ELISA kit served as an effective biomarker to distinguish CP from HV samples and from pancreatic ductal adenocarcinoma. However, many subjects exhibited levels below the level of detection of the PhoSL-HP assay; therefore, a highly sensitive detection method for PhoSL-HP is needed to evaluate PhoSL-HP as a potential pancreatic disease biomarker. Furthermore, it is also necessary to determine the utility of PhoSL-HP as a complement to CA19–9 for PC diagnosis through combination analysis. To address these issues, in the current study we developed a highly sensitive PhoSL-ELISA system to detect fucosylated haptoglobin using a high concentration of urea as a denaturing agent and tested its performance for PC discrimination.

## Material and methods

### Haptoglobin purification from conditioned medium

HepG2 human hepatocellular carcinoma cells (RIKEN Bio Resource Center, Ibaraki, Japan) were cultured in Dulbecco’s modified Eagle medium (Sigma Aldrich, St. Louis, MO, USA) supplemented with 10% fetal bovine serum at 37 °C in 5% CO_2_. After transfer into serum-free medium, the cells were cultured for 4 days. The conditioned medium was then harvested and used to isolate haptoglobin. The filtered medium was applied to an NHS-activated Sepharose 4 Fast Flow affinity column coupled with an anti-haptoglobin antibody (Medical & Biological Laboratories, Nagoya, Japan). After washing with 10 mM phosphate-buffered saline (PBS) with 1 M NaCl, the haptoglobin-bound fraction was eluted with 0.1 M glycine-HCl, pH 3.0 and immediately neutralized with 1 M Tris-HCl (pH 9.0) prior to concentration by ultrafiltration. An anti-haptoglobin antibody ELISA was then performed to evaluate the amount of purified haptoglobin according to a known haptoglobin standard (ERM-DA470, Sigma Aldrich). As AAL affinity chromatography (Online Resource Data 1) and MALDI-TOF mass spectrometry analysis confirmed that almost all haptoglobin from HepG2 medium contained core-fucosylated N-glycans (Online Resource Data 2), purified haptoglobin was defined as fucosylated haptoglobin and the PhoSL reactivity of 100 ng/mL fucosylated haptoglobin was defined as 100 mU/mL. AAL affinity chromatography was performed using an HPLC column (J-Oil Mills, Tokyo, Japan). Because AAL has a higher affinity for α1–6 linked than for α1–3 linked fucose, core fucosylated *N*-glycans were separated using an AAL-HPLC column. Mass spectra were analyzed using proprietary bioinformatics software from Sumitomo Bakelite (Tokyo, Japan).

### Lectin-antibody ELISA for fucosylated haptoglobin for PC detection (PhoSL-HP ELISA method 1)

The lectin-antibody ELISA for fucosylated haptoglobin (PhoSL-HP) was performed as described previously [11]. Briefly, a 96-well ELISA plate was coated with the Fab fragment of anti-human HP IgG (Dako, Carpinteria, CA, USA), because IgG has fucosylated oligosaccharides in its Fc portion. The coated plates were blocked with PBS containing 3% bovine serum albumin (BSA) for 1 h, followed by washing with PBS containing 0.1% Tween 20 (PBS-T). Purified haptoglobin from HepG2 medium (0–100 mU/mL, 50 μL) was added to each well and the plate was incubated for 1 h at room temperature (25 °C). The plate was then washed three times with PBS-T. Biotinylated PhoSL (J-Oil Mills) was added to each well and the plate was incubated for 30 min at 4 °C. After three further washes, peroxidase-conjugated streptavidin was added to each well, followed by incubation for 30 min at room temperature. After the plate was washed four times, 3, 3′, 5, 5′-tetramethylbenzidine (TMB) solution was added to each well and 0.5 M sulfuric acid was used to stop the reaction.

### Evaluation of the denaturing effect in the PhoSL-based ELISA (PhoSL-HP ELISA methods 2 and 3)

#### (PhoSL-HP ELISA method 2)

To develop an improved PhoSL-based ELISA, we evaluated candidates for the immobilized antibody (poly- and monoclonal antibodies). The mouse monoclonal antibody J101103 (J-Oil Mills) was selected as the immobilized antibody for the detection of PhoSL in the ELISA. A mouse monoclonal anti-human haptoglobin antibody digested with PNGaseF was used to coat a 96-well ELISA plate. The coated plates were blocked with PBS containing 1% BSA for 1 h, followed by washing PBS-T using an automatic plate washer (BioTec, Tokyo, Japan). After washing, the stabilizer StabilCoat (SurModics, Eden Prairie, MN, USA) was added and the plate was incubated for 30 min at room temperature (25 °C). Purified haptoglobin from HepG2 medium (0–100 mU/mL, 50 μL) was added to each well and the plate was incubated for 1 h at room temperature. The plate was then washed three times with PBS-T. Biotinylated PhoSL (J-Oil Mills) was added to each well and the plate was then incubated for 30 min at 4 °C. After three further washes, peroxidase-conjugated streptavidin was added to each well, followed by incubation for 30 min at room temperature. After the plate was washed four times, TMB solution was added to each well and 1 M phosphoric acid was used to stop the reaction.

#### (PhoSL-HP ELISA method 3)

To develop an improved PhoSL-based ELISA, we performed the PhoSL-ELISA and evaluated the effect of urea as follows. A mouse monoclonal anti-human haptoglobin antibody digested with PNGaseF was used to coat a 96-well ELISA plate. The coated plates were blocked with PBS containing 1% BSA for 1 h, followed by washing with PBS-T using an automatic plate washer (BioTec). After washing, StabilCoat was added and the plate was incubated for 30 min at room temperature (25 °C). Purified haptoglobin from HepG2 medium (0–100 mU/mL, 50 μL) was added to each well and the plate was incubated for 1 h at room temperature. The plate was then washed three times with PBS-T. Biotinylated PhoSL with 5 M urea was added to each well and the plate was then incubated for 30 min at 4 °C. After three further washes, peroxidase-conjugated streptavidin was added to each well, followed by incubation for 30 min at room temperature. After the plate was washed four times, TMB solution was added to each well and 1 M phosphoric acid was used to stop the reaction.

### Measurement of fucosylated haptoglobin in sera using the improved PhoSL-based ELISA

Serum samples from HVs were obtained from BIOPREDIC International (Rennes, France) and samples from patients with hepatocellular carcinoma (HCC), cholangiocarcinoma (CC), CP, and PC were obtained from ProteoGenex (Culver City, CA, USA) (Table 1). Serum sampling was carried out in accordance with standard operating procedures, which were consistent for both companies. The protocol for the human study was approved by the Institutional Review Boards of BIOPREDIC International and ProteoGenex Inc. The samples were collected after informed consent was obtained from these individuals. All samples were stored at less than −20 °C until use. The CA19–9 values were measured at Health Science Research Institute, Inc. (Kanagawa, Japan).

**Table 1:** Characteristics of the subjects in this study

	No.	Age (range, years)	Male	Female
Healthy volunteers (HVs)	40	55.7 (37–72)	25	15
Pancreatic cancer (PC)	34	59.4 (37–72)	14	20
Stage I	4	67.0 (63–72)	0	4
Stage II	9	58.6 (37–70)	2	7
Stage III	9	58.1 (52–70)	5	4
Stage IV	12	58.3 (37–71)	7	5
Hepatocellular carcinoma (HCC)	7	67.4 (54–77)	3	4
Cholangiocarcinoma (CC)	5	60.4 (47–73)	4	1
Chronic pancreatitis (CP)	24	59.0 (51–70)	9	15

The three methods for the PhoSL-HP ELISA were performed to quantitatively determine the serum core-fucosylated haptoglobin levels. The antibody-coated plate was constructed as described above. Conditioned media containing haptoglobin (0–140 mU/mL, 50 μL) [11], purified fucosylated haptoglobin from HepG2 medium (0–100 mU/mL, 50 μL), or 1:500 diluted serum were placed into each well, and the plate was incubated for 1 h at room temperature (25 °C) and then washed three times with PBS-T. For measurement of serum fucosylated haptoglobin, biotinylated PhoSL was diluted with or without 5 M urea with 0.1% Blockace (Megmilk Snow Brand, Tokyo, Japan) and 1% polyethylene glycol 200 in distilled water and added to each well, and the plate was then incubated for 30 min at 4 °C After the plate was washed three times, streptavidin and TMB reactions were performed as previously described.

Conditioned medium containing haptoglobin was used as a calibration standard for fucosylated haptoglobin in Method 1 (0–140 mU/mL, 50 μL) and purified fucosylated haptoglobin derived from HepG2 medium was used in Methods 2 and 3 (0–100 mU/mL). The concentrations of serum fucosylated haptoglobin were calculated using standard curves.

### Statistical analysis

The Steel-Dwass test was performed to identify statistically significant differences between groups. The correlations between the two marker units in sera were estimated based on Spearman’s correlation coefficient. The diagnostic performance of the scoring systems was assessed by analyzing receiver operating characteristic (ROC) curves. These tests were performed using R statistic software (https://www.r-project.org/).

## Results

### Establishment of the PhoSL-ELISA system using denaturation treatment

To improve the lectin reaction for the target glycans in haptoglobin, an immobilized mouse monoclonal antibody was selected for detection in the PhoSL-ELISA (data not shown). In addition, denaturation was performed using high-concentration urea in serum samples from HVs and patients with PC (Online Resource Data 3).

**Fig. 1:**
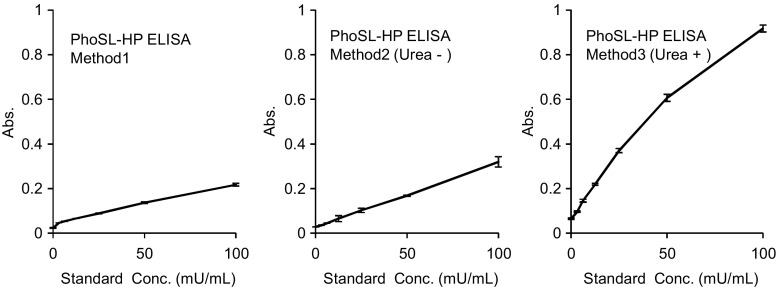
Measurement of standard haptoglobin using biotinylated PhoSL. (Method 1) Standard curve of the lectin-antibody ELISA using biotinylated PhoSL. The conditioned medium from a PC cell line, PK8, transfected with the haptoglobin expression vector was used as a standard using a previously reported procedure [11]. (Method 2) Standard curve of the lectin-antibody ELISA using biotinylated PhoSL. Purified haptoglobin from the conditioned medium of HepG2 cells was used as a standard. (Method 3) Standard curve of the lectin-antibody ELISA using biotinylated PhoSL under denaturing conditions. Purified haptoglobin from the conditioned medium of HepG2 cells was used as a standard

In the measurement of the standard sample (HepG2 haptoglobin), high absorbance values were obtained with the high-concentration urea treatment combined with PhoSL (Method 3), whereas PhoSL-HP Methods 1 and 2 showed a lower response than Method 3 (Fig. 1). The background signal did not differ among all conditions investigated. As shown in Online Resource Data 4, the correlation of PhoSL-HP values between our previous method (Method 1) and new method (Method 3) was very low. Accordingly, these results indicated that we succeeded in establishing a highly sensitive ELISA system to detect core-fucosylated haptoglobin in a limited fraction using a characteristic mouse monoclonal antibody, high-concentration urea, and PhoSL.

### Reevaluation of PhoSL-HP as a PC biomarker using the new system

**Fig. 2:**
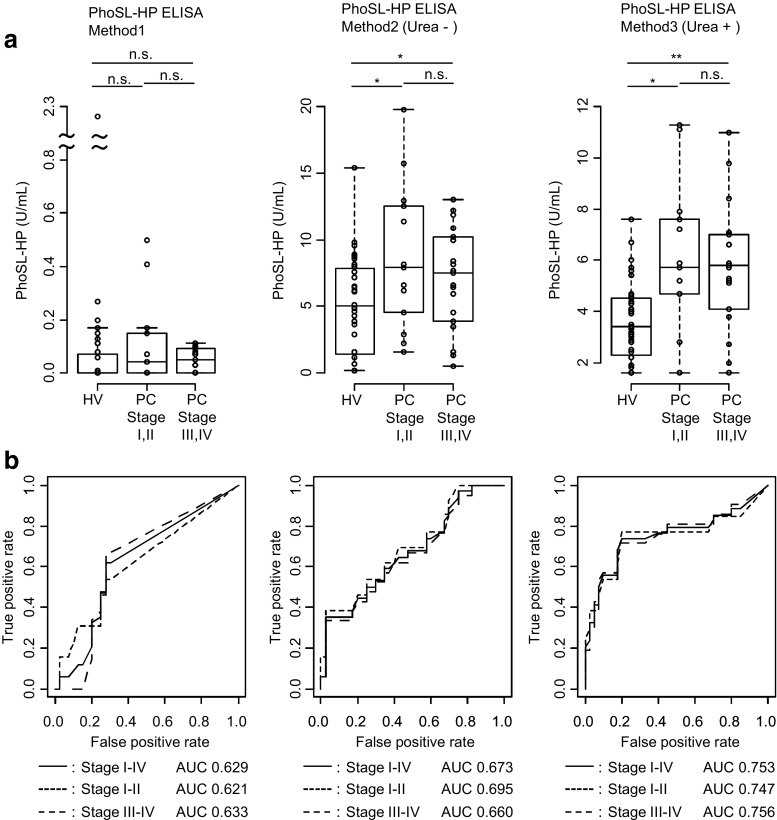
Serum levels of PhoSL-HP in healthy volunteers (HVs) and patients with pancreatic cancer (PC). **a** Boxplot analysis for the evaluation of PhoSL-HP as a PC biomarker using the previous PhoSL-ELISA system (Method 1) and a newly developed PhoSL-ELISA system (Methods 2 and 3). HVs (*n* = 40) and patients with PC in stage I (*n* = 4), stage II (*n* = 9), stage III (*n* = 9), and stage IV (*n* = 12) were investigated. The x-axis indicates the case classification, and the y-axis indicates the PhoSL-HP level (U/mL). Significant differences among the three states were determined using Steel-Dwass tests; *p < 0.05 and **p < 0.01. n.s., not significant. The boxes indicate interquartile ranges for each group of specimens. The bar represents the median value. **b** Receiver operating characteristic (ROC) and area under the curve (AUC) values for PhoSL-HP for distinguishing patients with PC from HVs (Method 3, a newly developed PhoSL-ELISA system)

Next, we reevaluated PhoSL-HP as a PC biomarker using our improved PhoSL-ELISA system by measuring PhoSL-HP levels in the sera of HVs and patients with PC (Fig. 2a). Steel-Dwass tests were performed to evaluate the differences among PhoSL-HP levels in each group (HVs or patients with PC in stages I/II or III/IV). PhoSL-HP levels in PC sera were higher than those in HV sera with all three methods (Fig. 2a). High absorbance values for PhoSL-HP were obtained with Method 3, whereas PhoSL-HP Methods 1 and 2 showed a lower response than Method 3. The use of a mouse monoclonal anti-human haptoglobin antibody and high-concentration urea treatment combined with PhoSL was the most effective method for precise measurement of PhoSL-HP in sera. The PhoSL-HP levels in patients with PC in stages I/II and III/IV were significantly different from those in HVs, whereas no significant difference was detected between stages I/II and III/IV in patients with PC.

To obtain a specificity of 80% or more, we established a cutoff value of 0.08 U/mL for PhoSL-HP in Method 1 based on ROC curve analysis. The positive rate (number) was 32% (11/34) in patients with PC and 20% (8/40) in HVs. The sensitivity of PhoSL-HP for PC was 32% (all stages) and the specificity was 80%. In comparison, we established a cutoff value of 5.7 U/mL for PhoSL-HP in Method 3 based on ROC curve analysis. The positive rate (number) was 56% (19/34) in patients with PC and 10% (4/40) in HVs. The sensitivity of PhoSL-HP for PC was 56% (all stages) and the specificity was 90%. PhoSL-HP (Method 3) (Urea +) was the most sensitive system. The ROC and area under the curve (AUC) values are shown in Fig. 2b. The AUC values for PhoSL-HP to distinguish patients with PC from HVs were highest in Method 3, being 0.747 (stages I/II), 0.756 (stages III/IV), and 0.753 (all stages).

In order to evaluate the specificity for PC of PhoSL-HP using Method 3(Urea+), PhoSL-HP levels were also examined in other related diseases including CP (Online Resource Data 5). The positive rates of PC stage I/II (54%) and stage III/IV (57%) were higher than those of HCC (29%), CC (20%), and CP (21%).

PC stage analysis of PhoSL-HP (Method 3) (Urea +) and CA19–9 were also performed (Online Resource Data 6). The serum levels of CA19–9 increased in each clinical stage. In contrast, the serum levels of PhoSL-HP were high in patients with PC at all clinical stages compared to the levels in HVs.

### Combined analysis with PhoSL-HP and CA19–9

**Fig. 3:**
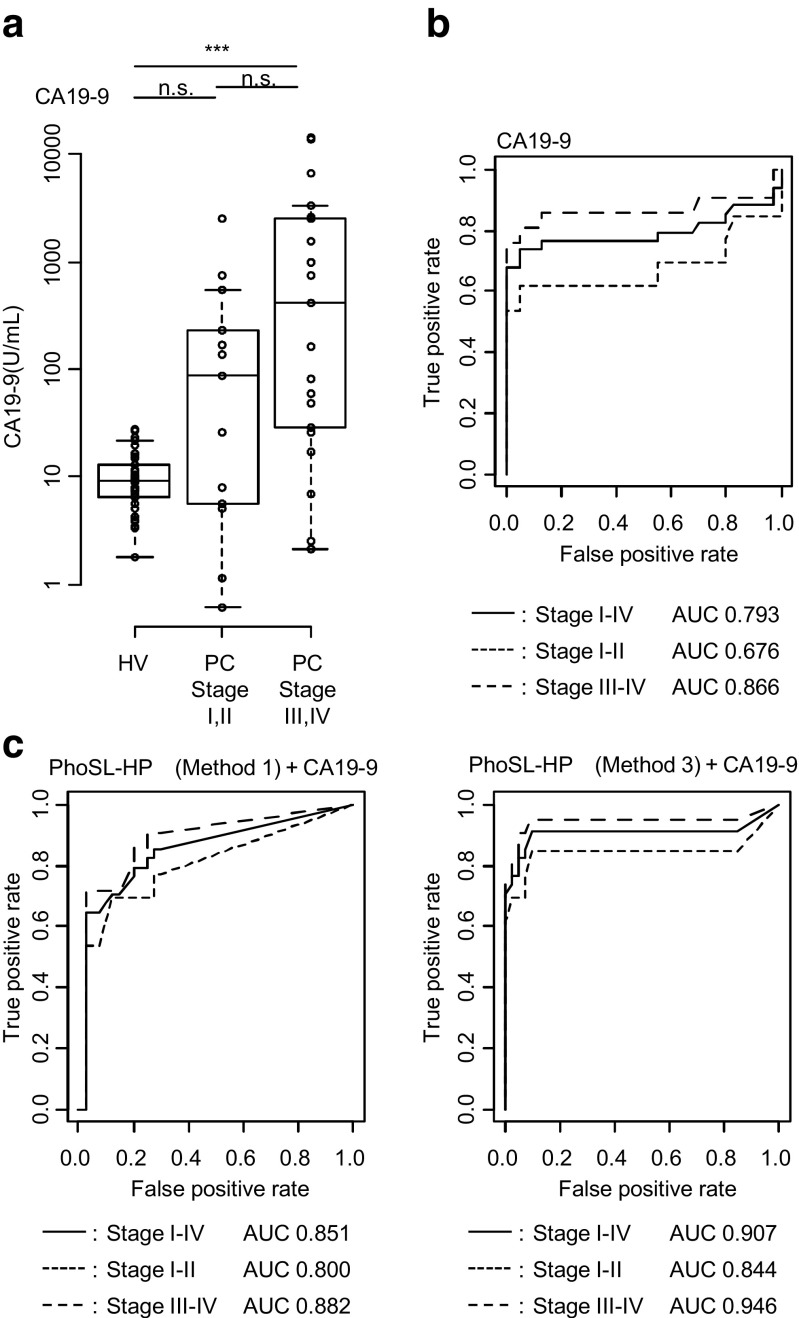
Efficacy of CA19–9 and PhoSL-HP for the diagnosis of pancreatic cancer (PC). **a** Boxplot analysis of CA19–9. Healthy volunteers (HVs) (*n* = 40) and patients with PC in stage I (*n* = 4), stage II (*n* = 9), stage III (*n* = 9), and stage IV (*n* = 12) were investigated. The x-axis indicates the case classification, and the y-axis indicates the CA19–9 level (U/mL). Significant differences among the three states were determined using Steel-Dwass tests; *p < 0.05, **p < 0.01, and ***p < 0.001. n.s., not significant. The boxes indicate interquartile ranges for each group of specimens. The bar represents the median value. **b** Receiver operating characteristic (ROC) curve and area under the curve (AUC) values for CA19–9. **c** ROC curve and AUC values for the PhoSL-HP and CA19–9 combined analysis for distinguishing patients with PC from HVs

CA19–9 levels were elevated in patients with PC at stages III/IV compared with the levels in HVs, although the difference between patients with PC at stages I/II and HVs was not significant (Fig. 3a). When the cutoff value was set to 37 U/mL for CA19–9, the sensitivity of CA19–9 was 54% (stages I/II), 71% (stages III/IV), and 65% (all stages), and the specificity was 100%.

ROC curves for PhoSL-HP and CA19–9 were constructed (Fig. 3b and c) and the AUC values for PhoSL-HP (Method 3) to distinguish patients with PC from HVs were 0.747 (stages I/II), 0.756 (stages III/IV), and 0.753 (all stages) (Fig. 2b). In contrast, the AUC values for CA19–9 were 0.676, 0.866, and 0.793, respectively (Fig. 3b). Accordingly, the performance of PhoSL-HP (Method 3) as a marker for PC in all stages was comparable to that of CA19–9; notably, however, in the case of early-stage patients, PhoSL-HP exhibited better performance. As shown in Online Resource Data 7, Spearman’s correlation analysis was used to determine the minimal correlation between the two markers, with a correlation coefficient of 0.154. A combined analysis with PhoSL-HP and CA19–9 was performed and an ROC curve was constructed (Fig. 3c). The AUC of CA 19–9 increased from 0.793 to 0.851 when combined with PhoSL-HP (Method 1), and to 0.907 when combined with PhoSL-HP (Method 3). After setting the cutoff values for PhoSL-HP (Method 3) and CA19–9 at 5.7 and 37 U/mL, respectively, the sensitivity for PC increased to 85% (stages I/II), 95% (stages III/IV), and 91% (all stages). Thus, the sensitivity of CA19–9 for detecting PC increased in combination with PhoSL-HP.

The ability of PhoSL-HP and CA19–9 to diagnose patients was also investigated. Among 34 patients with PC, 12 cases were diagnosed as CA19–9-negative, 9 of which were positive for PhoSL-HP (Table 2); of these 9 cases, 4 were in stage I or II (Table 2). Although the sample size was small, these results suggest that PhoSL-HP might be useful to diagnose early-stage PC in combination with CA19–9.

**Table 2:** Combined analysis with PhoSL-HP and CA19–9 in patients with PC

Patients (*n* = 34)		CA19–9 (Cut off: 37 U/mL)		Total
		True positive	False negative	
PhoSL-HP (Cut off: 5.7 U/mL)	True positive	10 (3*)	9 (4*)	19 (7*)
	False negative	12 (4*)	3 (2*)	15 (6*)
Total		22 (7*)	12 (6*)	34 (13*)

*Early-stage cases (stages I and II)

## Discussion

A new approach of detecting core-fucosylated haptoglobin in the sera of patients with PC was developed using an improved PhoSL-ELISA system. In previous studies, PhoSL-HP levels were found to be elevated in colorectal cancer and CP, whereas PhoSL-HP levels in many patients with PC were not detectable [11, 12]. However, there have also been some reports of elevated levels of core-fucosylated haptoglobin in the sera of patients with PC [7, 8, 14]. Because there are various HP forms in human sera, it is thought that this discrepancy is due to the type of anti-haptoglobin antibody used in each assay. To reconcile this problem, we developed a highly sensitive method to detect core-fucosylated haptoglobin captured with a monoclonal antibody; in particular, the use of high-concentration urea in this assay resulted in a drastic increase in the sensitivity for PhoSL-HP detection (Fig. 1). We screened potential antibodies using three characteristics: the binding ability, sensitivity of the standard curve, and measured values in serum samples (HVs and PC). A suitable antibody was identified after screening seven monoclonal antibodies and one polyclonal antibody that had been reported previously [11]. Our selected antibody exhibited a superior detection value (OD value) that was both high and stable. In addition, the coefficients of variation values for three replicate measures of the standards were within 10%. Although the background noise of this antibody was a little higher than that of a polyclonal antibody reported previously [11], there are several advantages to using monoclonal antibodies, such as production management. In addition, we also evaluated the use of a denaturing agent and demonstrated that urea proved to be the best agent to treat serum. Although the mechanism underlying this highly sensitive detection method remains unclear, the high concentration of urea likely induced conformational changes in the haptoglobin, providing enhanced exposure of the glycan sites to allow more effective interaction with PhoSL. Furthermore, although high-concentration urea is known to denature several types of proteins and can also decrease the biochemical activity of proteins, in the present study the binding affinity for PhoSL was maintained with high-concentration urea and higher absorbance values were observed, whereas the use of PhoSL-HP without urea failed to show high absorbance values (Fig. 1). This result indicates that PhoSL is highly resistant to denaturation agents. Notably, Kobayashi *et al.* [16] showed that PhoSL maintains its lectin activity under severe pH and temperature conditions; this feature might contribute to its resistance to high-concentration urea. As shown in Online Resource Data 4, there was no correlation observed between our previous method (Method 1) and the new method (Method 3). The lack of correlation may be attributable to the low measurement sensitivity of Method 1, because many specimens are below the minimum detection sensitivity using Method 1 as previously reported [12] compared to Method 3. Furthermore, the different antibodies used in Method 1 and Method 3 may also explain the lack of correlation. It is possible that the polyclonal antibody captures various types of antigen expressed on haptoglobin, whereas the monoclonal antibody captures only a specific antigen.

Here, we determined that the PhoSL-HP level was significantly higher in patients with PC compared to HVs (Fig. 2). Although PhoSL-HP levels of HCC, CC, and CP tended to be higher than those in HV samples, PC showed the highest levels compared to other related diseases (Online Resource Data 5). According to the ROC analysis, although the performance of PhoSL-HP alone was not superior to that of CA19–9, the AUC value of CA19–9 was greatly improved from 0.793 to 0.907 when combined with PhoSL-HP. (Fig. 3). The likely reason for this complementary effect was that these compounds recognize different target glycans. Consistent with this speculation, the correlation between the two markers was low, with a coefficient of 0.154. In addition, the positive rate for the PhoSL-HP level in stages I and II was high. The AUC-ROC results improved in the ROC analysis when measuring both PhoSL-HP and CA19–9.

Furthermore, measuring both CA19–9 and PhoSL-HP enabled the detection of 9 PC cases among 12 patients who were negative for CA19–9 alone (Table 2). CA19–9 is a sialyl Lewis^A^ antigen that is modified by the enzyme FUT3. Therefore, CA19–9 is ineffective for detecting PC in patients who lack this enzyme. However, core fucosylation is not influenced by FUT3; therefore, the detection of PhoSL-HP would not be influenced by the Lewis-negative phenotype, as is also the case for DUPAN-2 [3].

We also found that the median values of PhoSL-HP for patients with PC in stages I/II and III/IV were similar, whereas the median value of CA19–9 increased as the stage progressed. In the ROC analysis, the AUC values for PhoSL-HP in early-stage PC did not differ from the values for late-stage PC (Fig. 3). PC has one of the poorest prognoses of all cancers because most patients are diagnosed at advanced stages [18], during which resection is often impossible. In contrast, in the early stage when the tumor size is small (10 mm or less), the 5-year survival rate is significantly higher than that in cases with larger tumors [19]. Therefore, it is important to identify PC at an early stage. In the present study, the sensitivity of detecting PC in early-stage patients was greatly improved from under 60% when using PhoSL-HP or CA19–9 alone to 85% when using both PhoSL-HP and CA19–9. A combination analysis with the two markers might therefore be useful to diagnose PC in the early stages as well as the Lewis-negative phenotype. However, as the sample size was limited in the present study, further studies are necessary to investigate the characteristics of PhoSL-HP as a cancer biomarker.

In conclusion, in this study, we showed that PhoSL-HP might serve as a useful biomarker for PC in addition to the other biomarkers that are currently used in clinical practice. Future studies should elucidate the production mechanism and validate the clinical significance of PhoSL-HP in early-stage cancer diagnosis. Although further clinical research is required to evaluate the usefulness of PhoSL-HP as a PC biomarker, we anticipate that it will substantively contribute to the diagnosis of PC.

## Electronic supplementary material

Online Resource Data 1 AAL affinity chromatography analysis of α1–6 linked fucosylated *N*-glycans in haptoglobins. *N*-glycans were released with PNGaseF (Roche Applied Science, Penzberg, Germany). After using actinase E (Kaken Pharmaceutical, Tokyo, Japan), the digested fraction was mixed with 2 M acetic acid and incubated at 80 °C for 2 h to remove sialic acid. After desalting followed by evaporation, *N*-glycans were labeled with 2-aminopyridine (PA). *N*-glycans were purified using a Monospin-NH2 column (GL Science, Tokyo, Japan). Purified PA-labeled *N*-glycans were then subjected to AAL-HPLC to isolate α1–6-linked fucosylated *N*-glycans. HPLC analysis was performed on a Prominence System with an RF-20Axs fluorescence detector (Shimadzu). Separation was performed at 25 °C using stepwise conditions with 10 mM ammonium acetate (solvent A) and solvent B (5 mM L-fucose in solvent A). Stepwise elution was performed with 100% solvent B for 10 min. The flow rate was 0.8 mL/min. Detection was performed by fluorometry at an excitation wavelength of 310 nm and an emission wavelength of 380 nm. **a** Structures of PA-labeled *N*-glycan standard. **b** Chromatogram of standard mixture of PA-labeled *N*-glycans. Ten types of commercially available PA-labeled *N*-glycans (Takara Bio, Kyoto, Japan, and J-Oil Mills) were analyzed as *N*-glycan standards. **c** Chromatogram of *N*-glycans of HepG2 haptoglobin. (PDF 302 kb)

Online Resource Data 2 MALDI-TOF mass spectrometry analysis of *N*-glycan of HepG2 haptoglobin. **a** Stacked bar graph indicates the percentage of *N*-glycan types detected. **b** *N*-glycan compositions were assigned to each peak based on m/z, and structures were proposed by bioinformatics programs. The intensities were normalized to that of an internal standard with known concentration. (PDF 186 kb)

Online Resource Data 3 Effect of using urea to denature sera for the detection of fucosylated haptoglobin. To measure serum fucosylated haptoglobin, biotinylated PhoSL was diluted with a 0–10 M urea solution with 0.1% Blockace (Megmilk Snow Brand, Tokyo, Japan) and 1% polyethylene glycol 200 in distilled water. The black dots indicate the patients with pancreatic cancer, and the white dots indicate the normal volunteers. (PDF 180 kb)

Online Resource Data 4 Correlation of PhoSL-HP values with our previous method (Method 1) and a newly developed method (Method 3). The black dots indicate the patients with pancreatic cancer, and the white dots indicate the normal volunteers. (PDF 257 kb)

Online Resource Data 5 Boxplot analysis to verify cross-reactivity of PhoSL-HP (Method 3). Patients with HCC, CC, and CP were investigated, and the correlation between CA19–9 and PhoSL-HP (Method 3) was evaluated. (PDF 226 kb)

Online Resource Data 6 Boxplot analysis for the evaluation of PhoSL-HP as a pancreatic cancer (PC) biomarker using the newly developed PhoSL-ELISA system and CA19–9 with healthy volunteers (HVs) and patients with PC (stages I-IV). Comparison of the level of fucosylated haptoglobin and CA19–9 in HVs and patients with PC (stages I-IV) by boxplot analysis. The x-axis indicates the case classification and the y-axis indicates the PhoSL-HP level (U/mL) and CA19–9 level (U/mL). The boxes indicate the interquartile ranges for each group of specimens. The bar represents the median value. (PDF 240 kb)

Online Resource Data 7 Correlation of CA19–9 and PhoSL-HP (Method 3). The black dots indicate the patients with pancreatic cancer, and the white dots indicate the normal volunteers. (PDF 168 kb)

## Abbreviations

AAL: *Aleuria aurantia* lectin
AUC: Area under the curve
BSA: Bovine serum albumin
CA19–9: Carbohydrate antigen 19–9
CP: Chronic pancreatitis
DUPAN-2: Duke pancreatic monoclonal antigen type 2
ELISA: Enzyme-linked immunosorbent assay
HP: Haptoglobin
HVs: Healthy volunteers
PBS: Phosphate-buffered saline
PC: Pancreatic cancer
HCC: Hepatocellular carcinoma
CC: Cholangiocarcinoma
CP: Chronic pancreatitis
PhoSL: *Pholiota squarrosa* lectin
PhoSL-HP: *Pholiota squarrosa* lectin-reactive haptoglobin
ROC: Receiver operating characteristic
TMB: 3, 3′, 5, 5′-tetramethylbenzidine
