# Supplementary figures and images for: Reevaluation of Pholiota squarrosa lectin-reactive haptoglobin as a pancreatic cancer biomarker using an improved ELISA system
Source: Glycoconj J. 2017 Apr 28;34(4):537–44. doi: 10.1007/s10719-017-9772-9 (PMC5502058; doi:10.1007/s10719-017-9772-9)

( Online Data1)

a

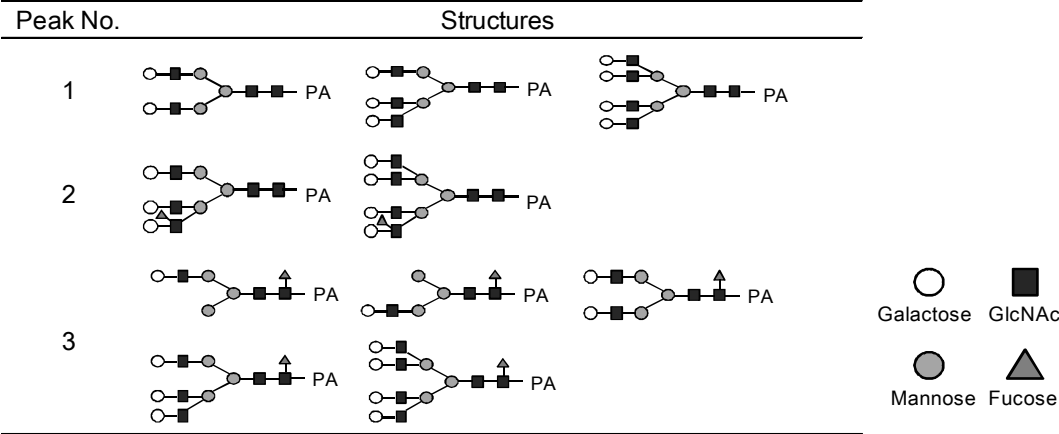

b

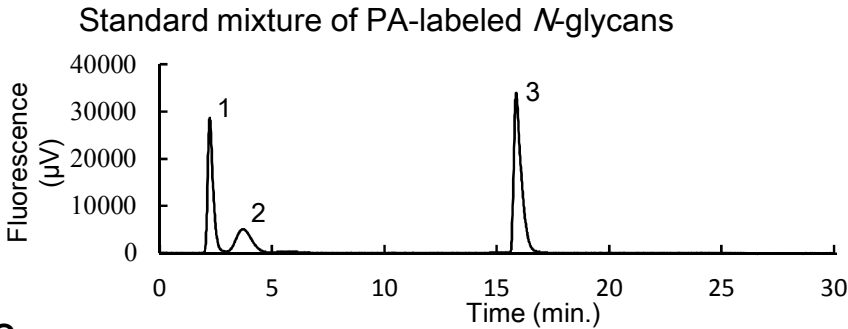

c

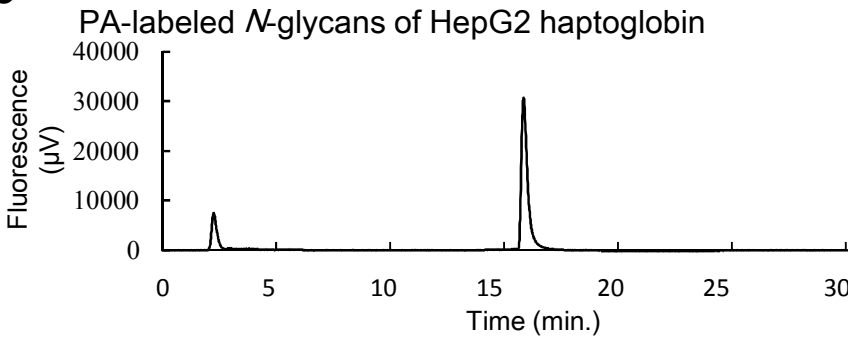

Supplement: Supplementary file 1 — AAL affinity chromatography analysis of α1–6 linked fucosylated N-glycans in haptoglobins. N-glycans were released with PNGaseF (Roche Applied Science, Penzberg, Germany). After using actinase E (Kaken Pharmaceutical, Tokyo, Japan), the digested fraction was mixed with 2 M acetic acid and incubated at 80 °C for 2 h to remove sialic acid. After desalting followed by evaporation, N-glycans were labeled with 2-aminopyridine (PA). N-glycans were purified using a Monospin-NH2 column (GL Science, Tokyo, Japan). Purified PA-labeled N-glycans were then subjected to AAL-HPLC to isolate α1–6-linked fucosylated N-glycans. HPLC analysis was performed on a Prominence System with an RF-20Axs fluorescence detector (Shimadzu). Separation was performed at 25 °C using stepwise conditions with 10 mM ammonium acetate (solvent A) and solvent B (5 mM L-fucose in solvent A). Stepwise elution was performed with 100% solvent B for 10 min. The flow rate was 0.8 mL/min. Detection was performed by fluorometry at an excitation wavelength of 310 nm and an emission wavelength of 380 nm. a Structures of PA-labeled N-glycan standard. b Chromatogram of standard mixture of PA-labeled N-glycans. Ten types of commercially available PA-labeled N-glycans (Takara Bio, Kyoto, Japan, and J-Oil Mills) were analyzed as N-glycan standards. c Chromatogram of N-glycans of HepG2 haptoglobin. (PDF 302 kb) [file 10719_2017_9772_MOESM1_ESM.pdf]

( Online Data3)

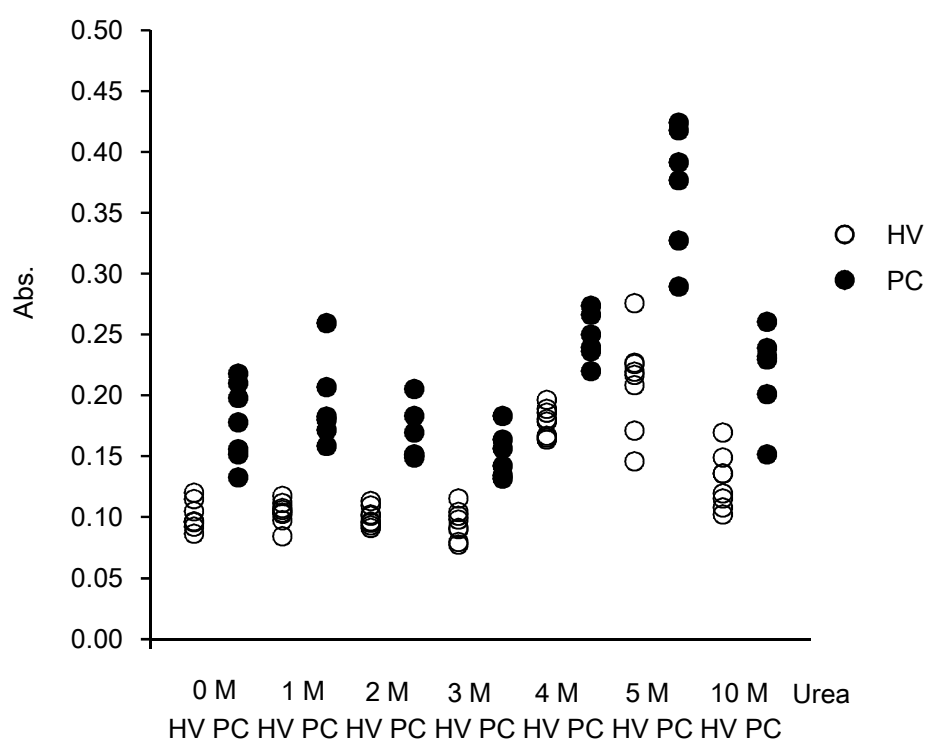

Supplement: Supplementary file 3 — Effect of using urea to denature sera for the detection of fucosylated haptoglobin. To measure serum fucosylated haptoglobin, biotinylated PhoSL was diluted with a 0–10 M urea solution with 0.1% Blockace (Megmilk Snow Brand, Tokyo, Japan) and 1% polyethylene glycol 200 in distilled water. The black dots indicate the patients with pancreatic cancer, and the white dots indicate the normal volunteers. (PDF 180 kb) [file 10719_2017_9772_MOESM3_ESM.pdf]

( Online Data4)

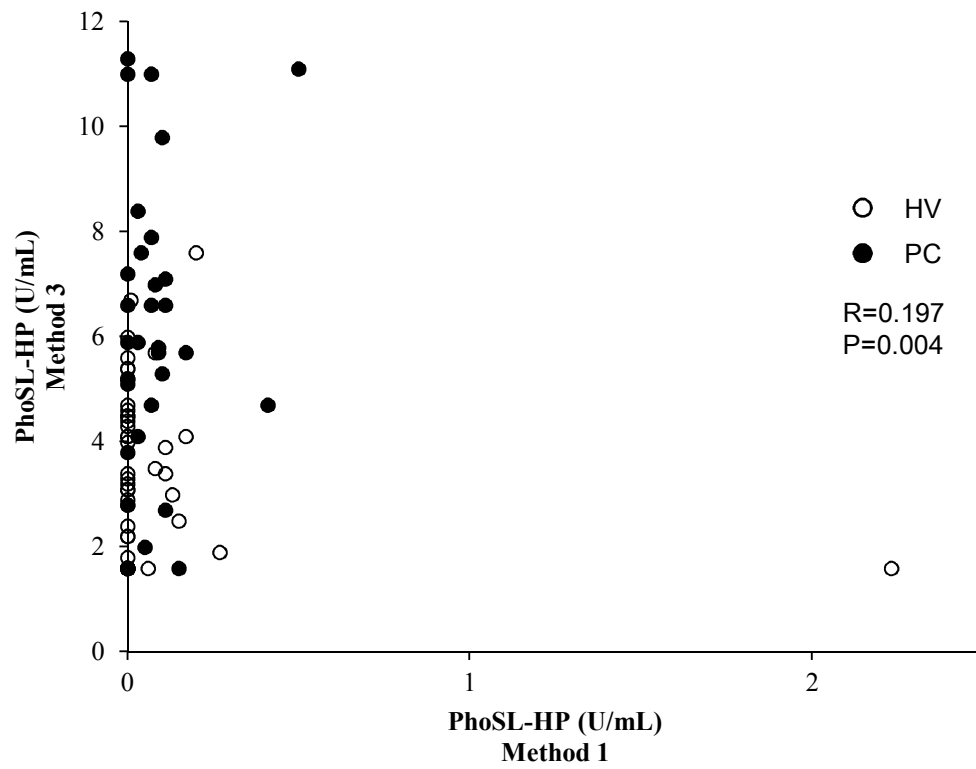

Supplement: Supplementary file 4 — Correlation of PhoSL-HP values with our previous method (Method 1) and a newly developed method (Method 3). The black dots indicate the patients with pancreatic cancer, and the white dots indicate the normal volunteers. (PDF 257 kb) [file 10719_2017_9772_MOESM4_ESM.pdf]

( Online Data5)

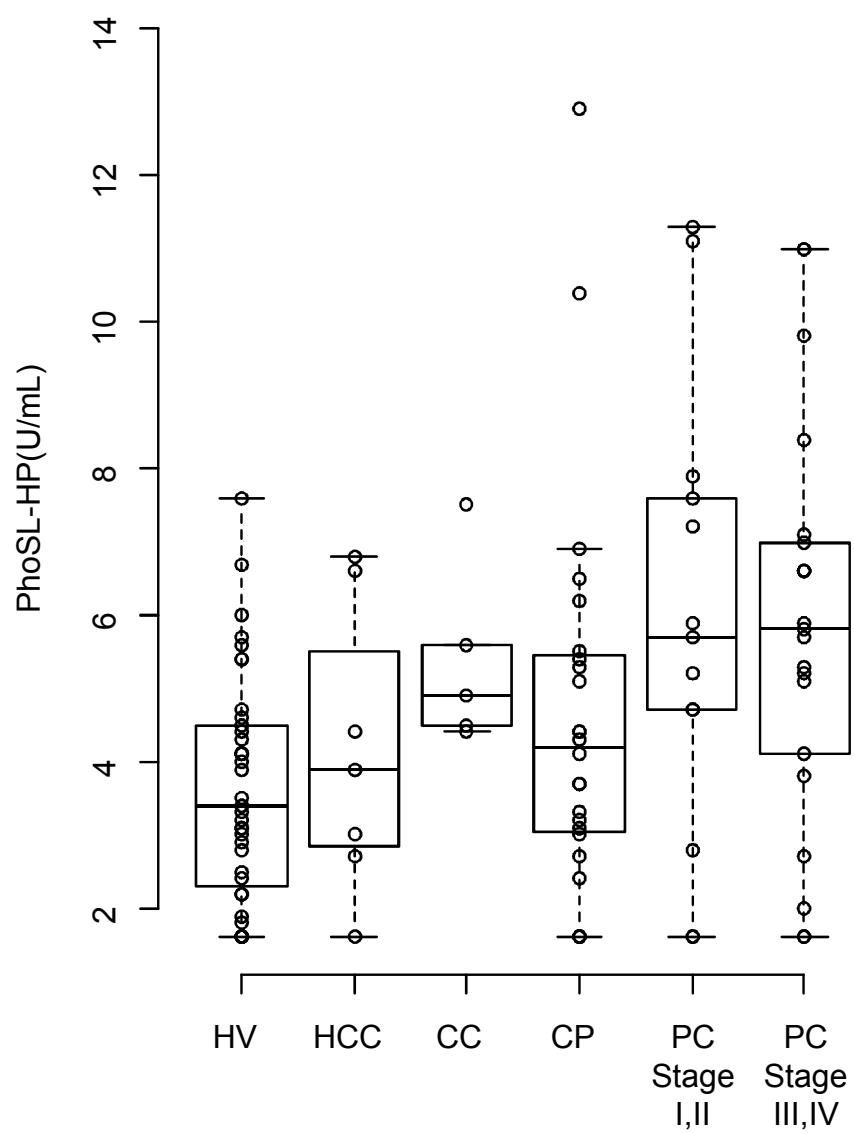

Supplement: Supplementary file 5 — Boxplot analysis to verify cross-reactivity of PhoSL-HP (Method 3). Patients with HCC, CC, and CP were investigated, and the correlation between CA19–9 and PhoSL-HP (Method 3) was evaluated. (PDF 226 kb) [file 10719_2017_9772_MOESM5_ESM.pdf]

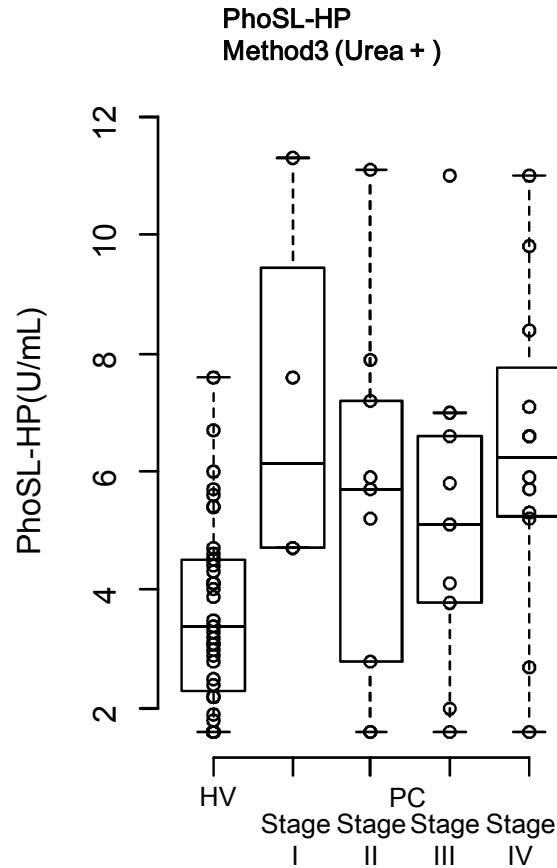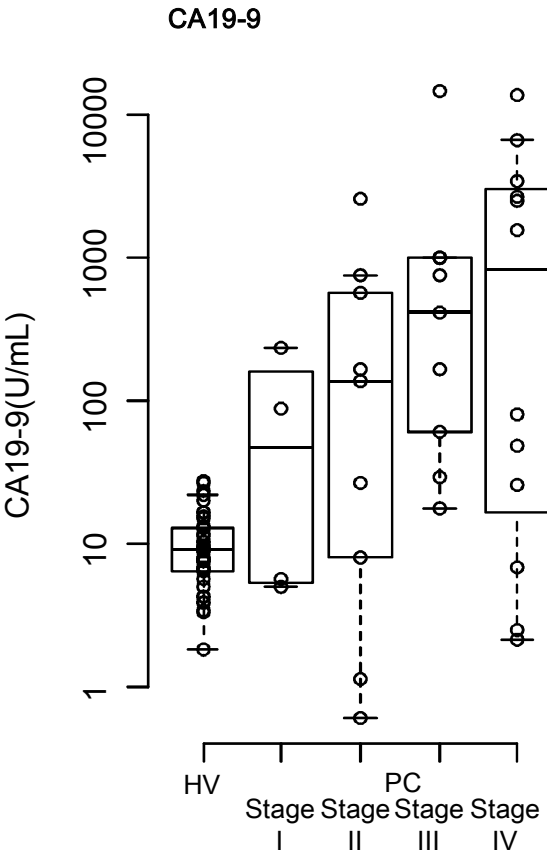

Supplement: Supplementary file 6 — Boxplot analysis for the evaluation of PhoSL-HP as a pancreatic cancer (PC) biomarker using the newly developed PhoSL-ELISA system and CA19–9 with healthy volunteers (HVs) and patients with PC (stages I-IV). Comparison of the level of fucosylated haptoglobin and CA19–9 in HVs and patients with PC (stages I-IV) by boxplot analysis. The x-axis indicates the case classification and the y-axis indicates the PhoSL-HP level (U/mL) and CA19–9 level (U/mL). The boxes indicate the interquartile ranges for each group of specimens. The bar represents the median value. (PDF 240 kb) [file 10719_2017_9772_MOESM6_ESM.pdf]

( Online Data7)

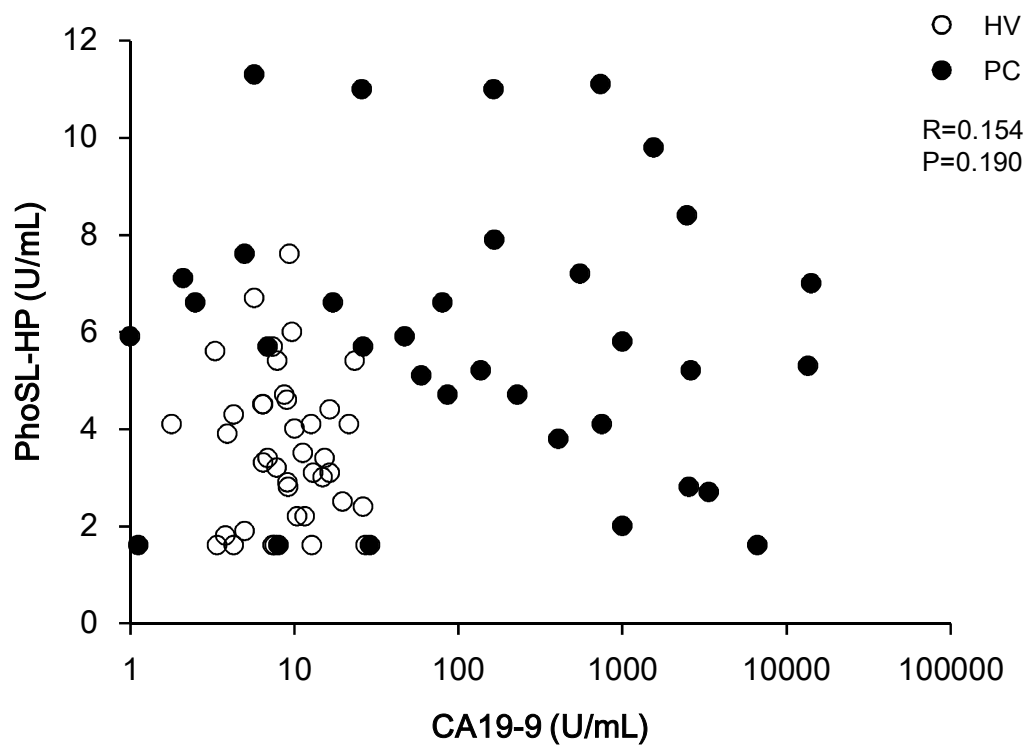

Supplement: Supplementary file 7 — Correlation of CA19–9 and PhoSL-HP (Method 3). The black dots indicate the patients with pancreatic cancer, and the white dots indicate the normal volunteers. (PDF 168 kb) [file 10719_2017_9772_MOESM7_ESM.pdf]
