# Supplementary material for: Reevaluation of Pholiota squarrosa lectin-reactive haptoglobin as a pancreatic cancer biomarker using an improved ELISA system
Source: Glycoconj J. 2017 Apr 28;34(4):537–44. doi: 10.1007/s10719-017-9772-9 (PMC5502058; doi:10.1007/s10719-017-9772-9)
Supplement: Supplementary file 2 — MALDI-TOF mass spectrometry analysis of N-glycan of HepG2 haptoglobin. a Stacked bar graph indicates the percentage of N-glycan types detected. b N-glycan compositions were assigned to each peak based on m/z, and structures were proposed by bioinformatics programs. The intensities were normalized to that of an internal standard with known concentration. (PDF 186 kb) [file 10719_2017_9772_MOESM2_ESM.pdf]

(Online Data2)

a

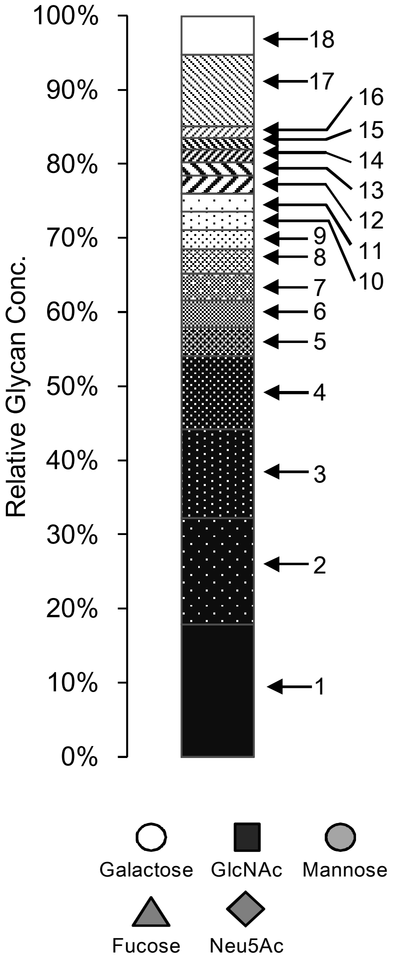

b

No.	m/z	Proposed structures	No.	m/z	Proposed structures
1	2950.07	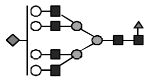	10	2890.05	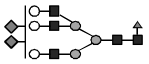
2	3096.13	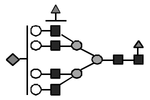	11	2584.94	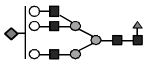
3	3401.24	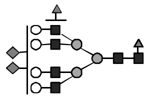	12	3560.30	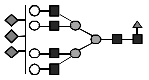
4	3255.18	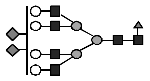	13	2731.00	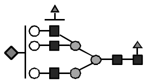
5	3242.19	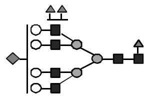	14	3195.16	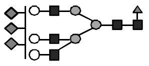
6	3706.35	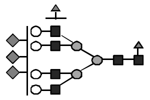	15	3852.39	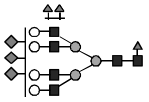
7	3547.27	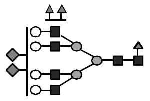	16	2524.92	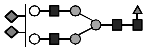
8	3036.11	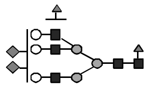	17	-	Other structeres including α1-6 linked fucose
9	3341.22	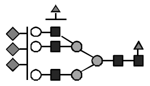	18	-	Other structres not containing fucose
